# Supplementary material for: Potent AMA1-specific human monoclonal antibody against Plasmodium vivax Pre-erythrocytic and Blood Stages
Source: Nat Commun. 2024 Dec 4;15:10556. doi: 10.1038/s41467-024-53848-4 (PMC11618605; doi:10.1038/s41467-024-53848-4)
Supplement: Supplementary file 1 — Supplementary Information [file 41467_2024_53848_MOESM1_ESM.pdf]

Supplemental Material

Potent AMA1-specific human monoclonal antibody against *Plasmodium vivax* Pre-erythrocytic and Blood Stages

Anna C. Winnicki [1,+], Melanie H. Dietrich [2,3,+], Lee M. Yeoh [4,5], Lenore L. Carias [1], Wanlapa Roobsoong [6], Chiara L. Drago [4,7], Alyssa N. Malachin [1], Karli R. Redinger [1], Lionel Brice Feufack-Donfack [8], Lea Baldor [8], Nicolai C. Jung [2], Olivia S. McLaine [1], Yelenna Skomorovska-Prokvolit [1], Agnes Orban [8], D. Herbert Opi [4,9,10], Payton Kirtley [11], Kiersey Nielsen [11], Maya Aleshnick [11], Gigliola Zanghi [12], Nastaran Rezakhani [12], Ashley M. Vaughan [12,13], Brandon K. Wilder [11], Jetsumon Sattabongkot [6], Wai-Hong Tham [2,3], Jean Popovici [8], James G. Beeson [4,9,10], Jürgen Bosch*[1,14], and Christopher L. King*[1,15]

Author information
[+] These authors contributed equally: Anna C. Winnicki and Melanie H. Dietrich
* These authors jointly supervised this work: Jürgen Bosch and Christopher L. King

Corresponding Authors: Jürgen Bosch and Christopher L. King
Emails: jbosch@case.edu and cxk21@case.edu

[1] Center for Global Health and Diseases, Department of Pathology, Case Western Reserve University School of Medicine, [2] Walter and Eliza Hall Institute of Medical Research, Parkville, Victoria, Australia, [3] Department of Medical Biology, The University of Melbourne, Parkville, Victoria, Australia, [4] Burnet Institute, Melbourne, Victoria, Australia, [5] Department of Medicine, The University of Melbourne, Parkville, Victoria, Australia, [6] Mahidol Vivax Research Unit, Faculty of Tropical Medicine, Mahidol University, Bangkok, Thailand, [7] Department of Microbiology, Monash University, Clayton, Victoria, Australia, [8] Malaria Research Unit, Institut Pasteur du Cambodge, Phnom Penh, Cambodia, [9] Department of Infectious Diseases, The University of Melbourne, Parkville, Victoria, Australia, [10] Central Clinical School and Department of Microbiology, Monash University, Clayton, Victoria, Australia, [11] Vaccine and Gene Therapy Institute, Oregon Health & Science University, Beaverton, OR, [12] Center for Global Infectious Disease Research, Seattle Children's Research Insititute, Seattle, WA, [13] Department of Pediatrics, University of Washington, Seattle, WA, [14] InterRayBio LLC, [15] Veterans Affairs Medical Center, Cleveland, OH

<u>Index Supplemental</u>

Figures:

Supplemental Figure 1 – Blocking activity of serum from Cambodian individuals
Supplemental Figure 2 – Flow diagram depicting PvAMA1-specific B cells that were isolated
Supplemental Figure 3 – HumAb Single Cycle Kinetics
Supplemental Figure 4 - Replicates of Pf-PvAMA1 cell line inhibition
Supplemental Figure 5 – Sporozoite HC04 invasion separated CSP210/247
Supplemental Figure 6 – Microscopic assessment of P. vivax liver in FRGN huHep mice after administration of anti-AMA1 human monoclonal antibody 826827
Supplemental Figure 7 - Surface properties of PvAMA1 and its interaction partners RON2 and humAb 826827
Supplemental Figure 8 – RON2 binding groove conservation and Sequence differences between Pv, PvPNG16, Pf, Pc, Pk, Tg
Supplemental Figure 9 – Published PvAMA1 Clinical Isolates sequence conservation
Supplemental Figure 10 - Epitope and paratope of the PvAMA1-826827 complex
Supplementary Figure 11 - Representative 2Fo-Fc electron density map of the PvAMA1-826827 crystal structure

Tables:

Supplemental Table 1 – VDJ alleles and CDR3 sequences and clonal groups
Supplemental Table 2 – Avidity Index (AI_{50}) of PvAMA1 specific humAbs to different AMA1 constructs
Supplemental Table 3 - List of SNP and haplotypes observed in AMA1 sequences in isolates tested in response to 826827
Supplemental Table 4 – Crystallography data collection and refinement statistics
Supplemental Table 5 - Interactions PvAMA1 – Fab 826827 based on PISA

Supplemental Tables & Figures

Supplemental Figure 1 – Blocking activity of serum from Cambodian individuals

Inhibition of PvRON2 binding to PvAMA1 by plasma samples from Cambodian donors. Plasma samples were tested at 1/50 dilution for the ability to inhibit binding of the PvRON2 loop to PvAMA1 in a plate-based assay. Samples were tested in duplicate. Data show mean and range; the dotted line shows 50% inhibition. PBMCs from donor C5 were selected for sorting B cells specific for PvAMA1 and subsequent MAb generation. Error bars show +/- SEM.

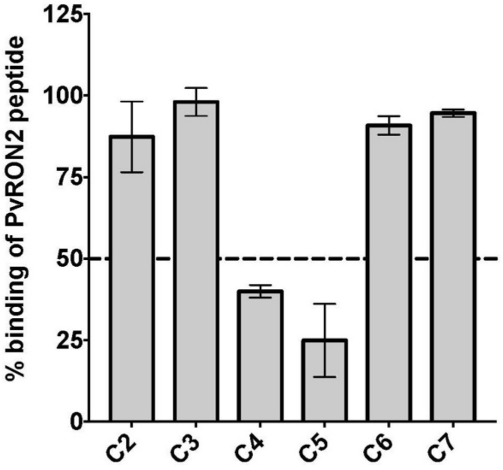

<u>Supplemental Figure 2. Representative Flow diagram depicting PvAMA1-specific B cells that were isolated</u>.

B cells were enriched using immunomagnetic positive selection with anti-CD19 magnetic MACS beads (Miltenyi Biotec, upper left panel). SYTOX Green Dead Cell Stain (Invitrogen) was used to gate out dead cells (lower left panel). Doublet discrimination was performed to exclude aggregated cells (upper middle panel) and stained with mouse anti-human CD20 (PE-Cy5.5; Invitrogen) and anti-human IgG Abs (PE-Cy7 clone G18-145; Becton Dickinson) to identify IgG expressing B cell (lower middle panel). Pv AMA1-specific B cells were identified using biotinylated PvAMA1 using Streptavidin coupled with Fluorescein isothiocyanate (FITC) or Brilliant Violet 421 (right panel showing sort gate). PvAMA1-cells were sorted on a BD FACSAria II.

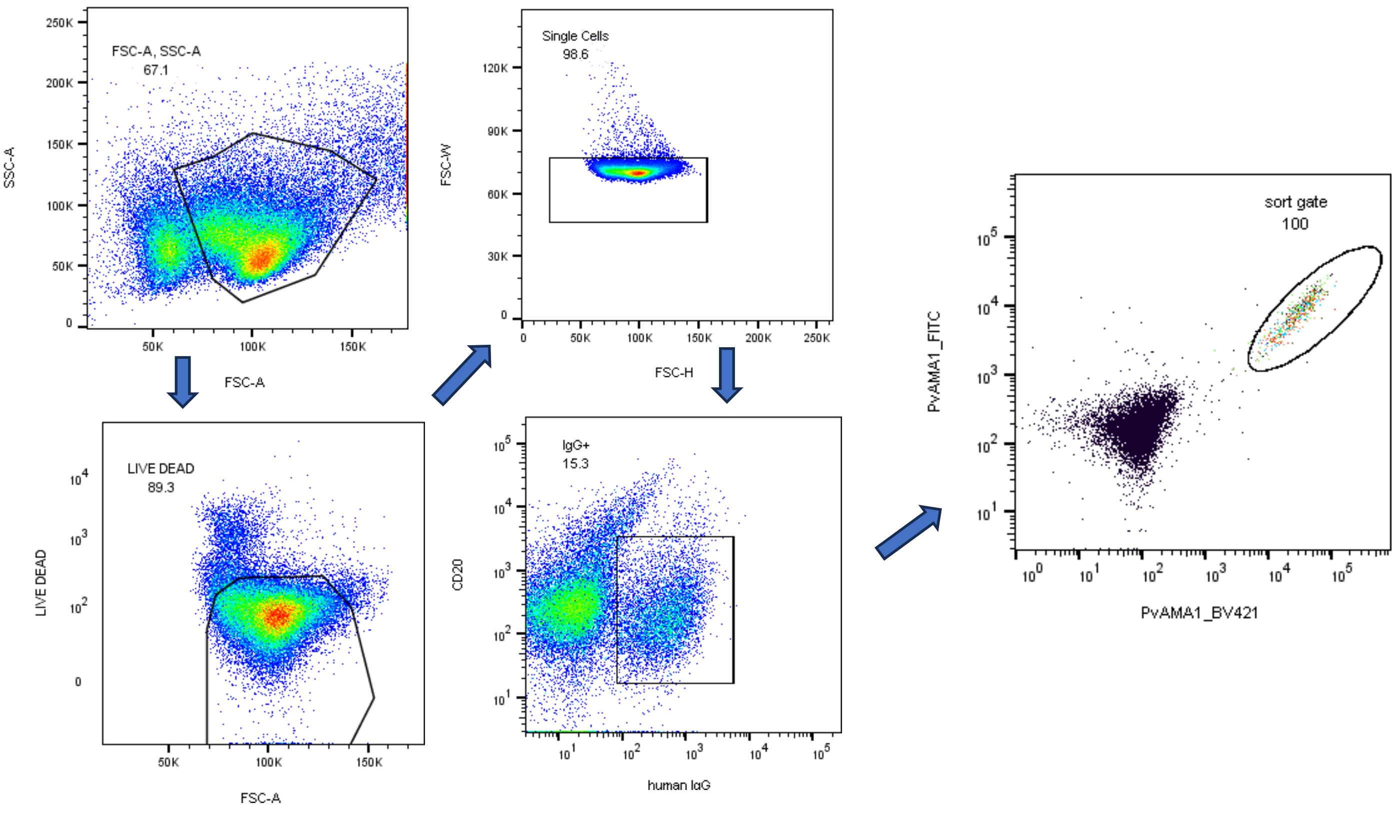

Supplement Figure 3 – HumAb Single Cycle Kinetics Curves and Results

A) Binding response curves to various concentrations of PvAMA1 for each humAb. **B)** k_{on} and k_{off} rates for each humAb determined using SPR.

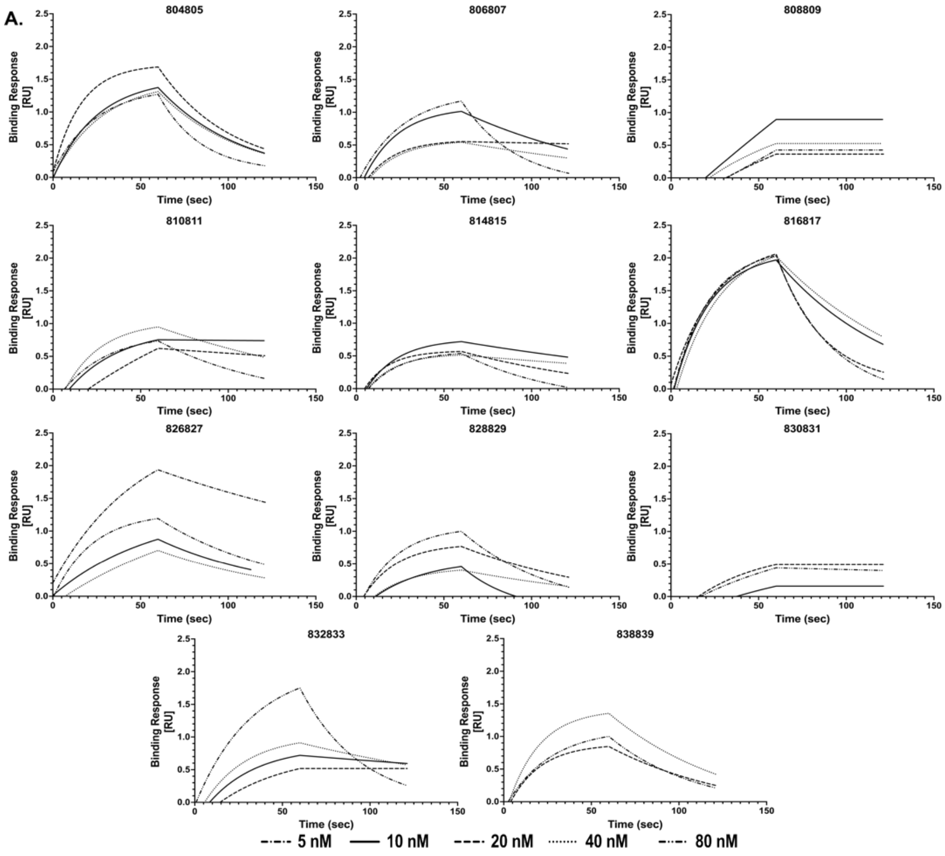

B.

	Kon	Koff
800801	0.00×10^{0}	0.00×10^{0}
804805	2.48×10^{6}	1.14×10^{-2}
806807	1.35×10^{6}	7.40×10^{-3}
808809	6.01×10^{5}	-2.35×10^{-3}
810811	8.64×10^{5}	4.44×10^{-3}
814815	2.56×10^{6}	5.30×10^{-3}
816817	1.78×10^{6}	1.46×10^{-2}
826827	6.01×10^{5}	1.02×10^{-2}
828829	1.28×10^{6}	1.32×10^{-2}
830831	1.01×10^{6}	-2.60×10^{-4}
832833	4.77×10^{5}	8.85×10^{-3}
838839	1.15×10^{6}	1.42×10^{-2}

Supplemental Figure 4 - Replicates of Pf-PvAMA1 cell line inhibition

Dose response inhibition curves of PvAMA1-specific humAbs against Pf-PvAMA1 transgenic parasites. Each humAb was tested in triplicate as indicated in different colored lines, and IC_{50} was calculated using R. 043038, an anti-tetanus toxoid humAb was used as a negative control.

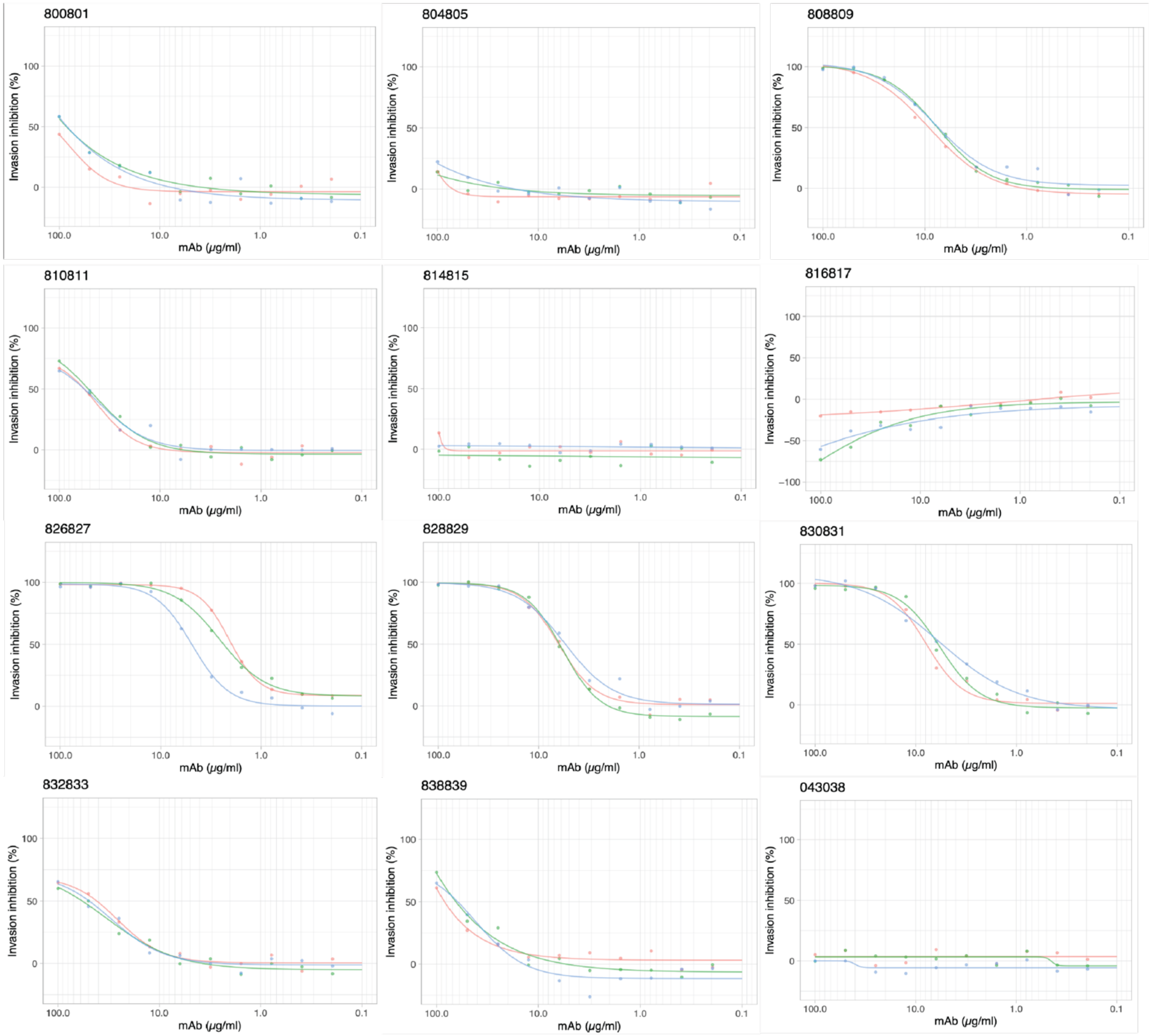

Supplemental Figure 5 – Sporozoite HC04 invasion separated CSP210/247

Dose response inhibition curves of Pv sporozoites blocking by PvAMA1 specific humAbs. Murine anti-CSP monoclonal antibodies served as positive control of blocking inhibition. Three different sporozoite isolates were used for this assay. Based on the blocking activity with the anti-CSP monoclonal one can separate two CSP210 and one CSP247 experiments. Shown below in **A)** are the dose-responses obtained with CSP210 strains and in **B)** with strain CSP247. The calculated IC 50 for CSP210 is 0.08 μg/mL and for CSP247 IC 50 ~8 μg/mL HumAbs against PvAMA1 were randomly screened with these isolates. Of note, humAb8 26827

was screened with both sporozoite strains and shows potent inhibition in both in contrast to the CSP monoclonal.

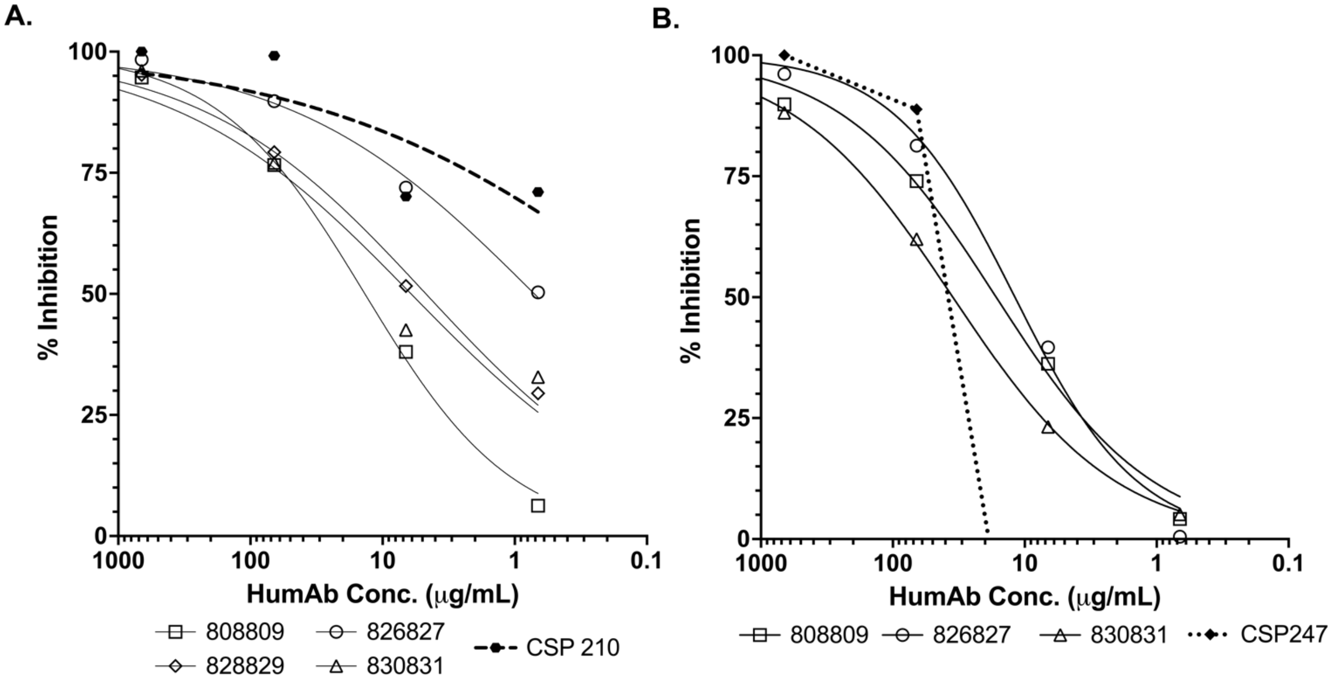

Supplemental Figure 6 – *Microscopic assessment of P. vivax liver in FRGN huHep mice after administration of anti-AMA1 human monoclonal antibody 826827.* The experimental design was identical to that described in the legend of Figure 4. On day 9, liver sections were analyzed microscopically for the presence of parasites described by (Mikolajczak et al. CHM, 2015). Both hypnozoites and schizonts were observed. This analysis shows total parasite forms in the liver (hypnozoites plus schizonts). Of note, one of the control livers could not be adequately evaluated and was not included in the analysis. Each dot represents one mouse. Shown in mean ± SD. Statistics: unpaired t-test.

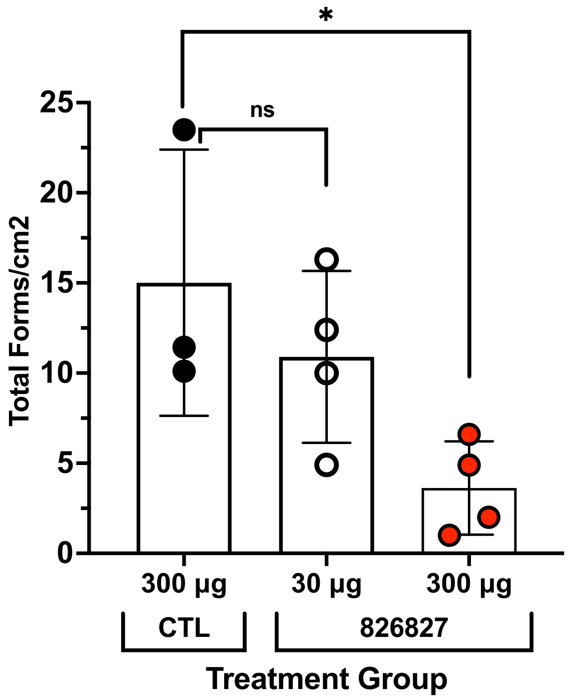

Supplemental Figure 7 - Surface properties of PvAMA1 and its interaction partners RON2 and humAb 826827

A) Schematic overview indicating the Domain 1 of PvAMA1 in gray with the Domain 2 loop in golden and the bound CDR3 loop of 826 in red. **B)** Hydrophobic surface potential of the PvAMA1 binding site. 826 was removed and rotated by 180˚ compared to the orientation in A to show the corresponding bottom interface of the interaction as well as the corresponding PvRON2 peptide. Darker areas represent higher hydrophobicity. Blue areas show hydrophilicity. **C)** Surface potential of the binding site. Red indicates negatively charged areas. Blue indicates positively charged areas. Figures were generated with Vida 4.4 from OpenEye.

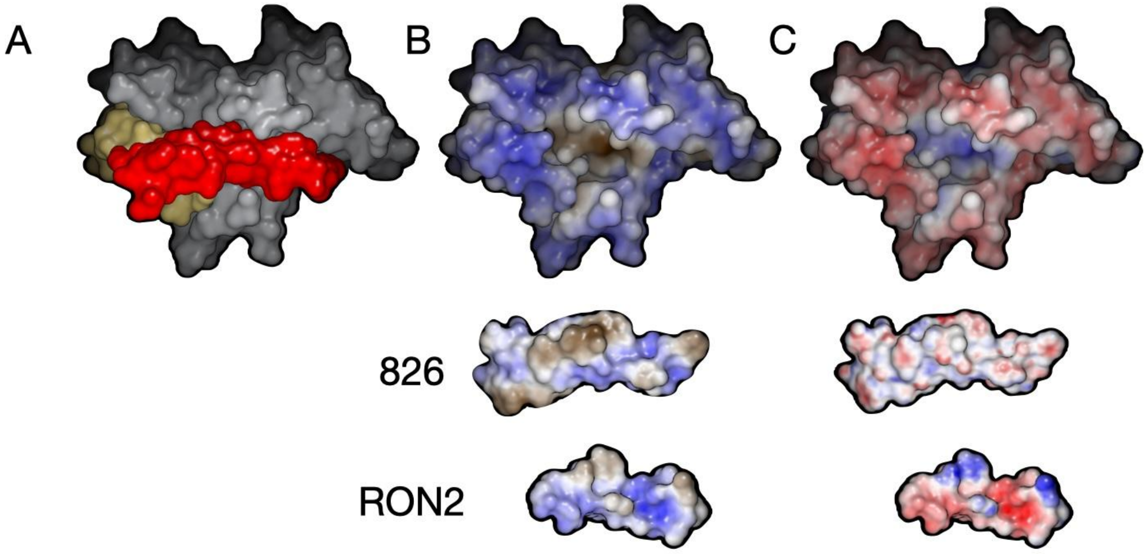

Supplemental Figure 8 – RON2 binding groove conservation and Sequence differences between Pv, PvPNG16, Pf, Pc, Pk, Tg

For all images, a residue-colored orange indicates an amino acid change between PvAMA1_PaloAlto (PDB: 9DX6) and another species' version of AMA1 **A)** Sequence conservation of residues surrounding the RON2 binding groove across 390 published clinical isolates **B)** PvAMA1_PaloAlto versus PvAMA1_PNG16 **C)** PvAMA1_PaloAlto versus *P. cynomolgi* **D)** PvAMA1_PaloAlto versus *P. knowlesi* **E)** PvAMA1_PaloAlto versus *P. falciparum* **F)** PvAMA1_PaloAlto versus *Toxoplasma gondii*.

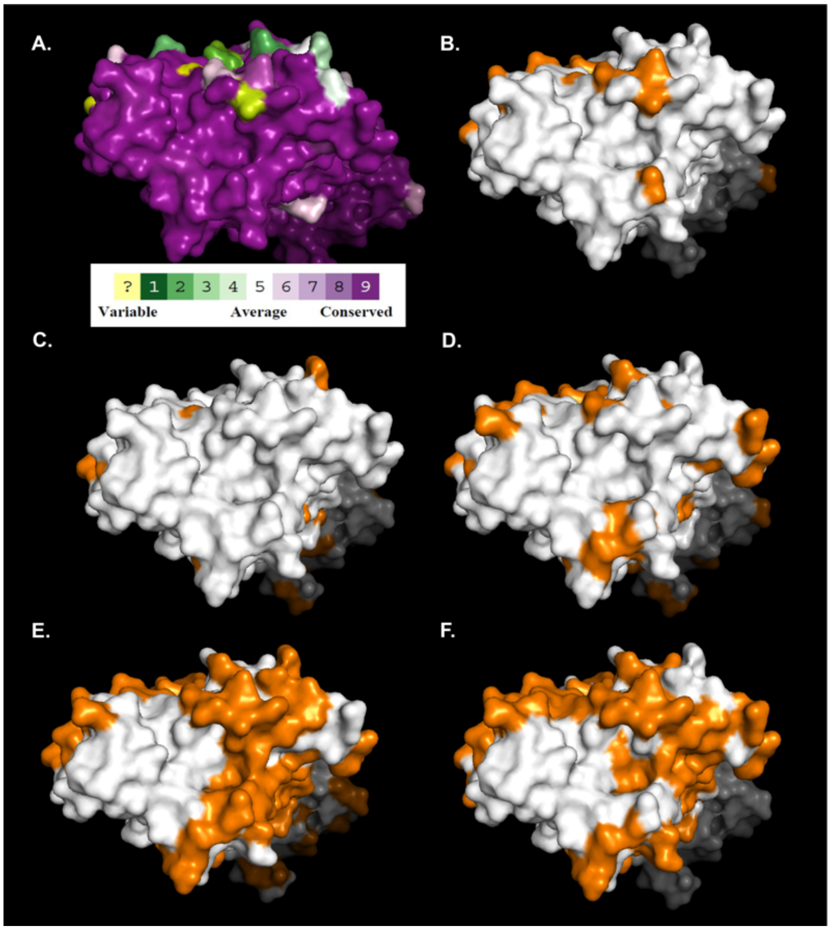

Supplemental Figure 9 – Published PvAMA1 clinical isolates sequence conservation

A) Percent conservation of each amino acid of PvAMA1 across 390 published clinical isolates. Yellow circles represent Domain 1 residues. Orange triangles represent Domain 2 residues. Blue squares represent Domain 3 residues. **B)** Percent conservation of PvAMA1 residues that contact the RON2 extracellular loop.

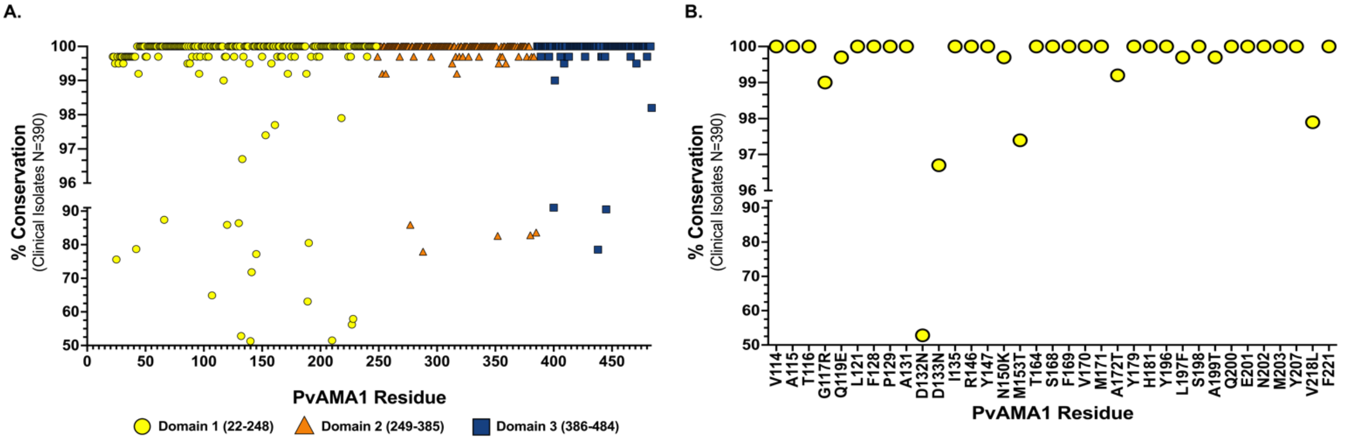

Supplementary Figure 10. Epitope and paratope of the PvAMA1-826827 complex.
(A) Surface representation of PvAMA1 with the epitope recognized by 826827 colored in yellow (LC contribution) and orange (HC contribution). (B) Surface representation of humAb 826827 with the paratope of PvAMA1 colored light blue (domain 1 contribution) and blue (domain 2 loop contribution). Residues within a distance cut-off of 5 Å are highlighted.

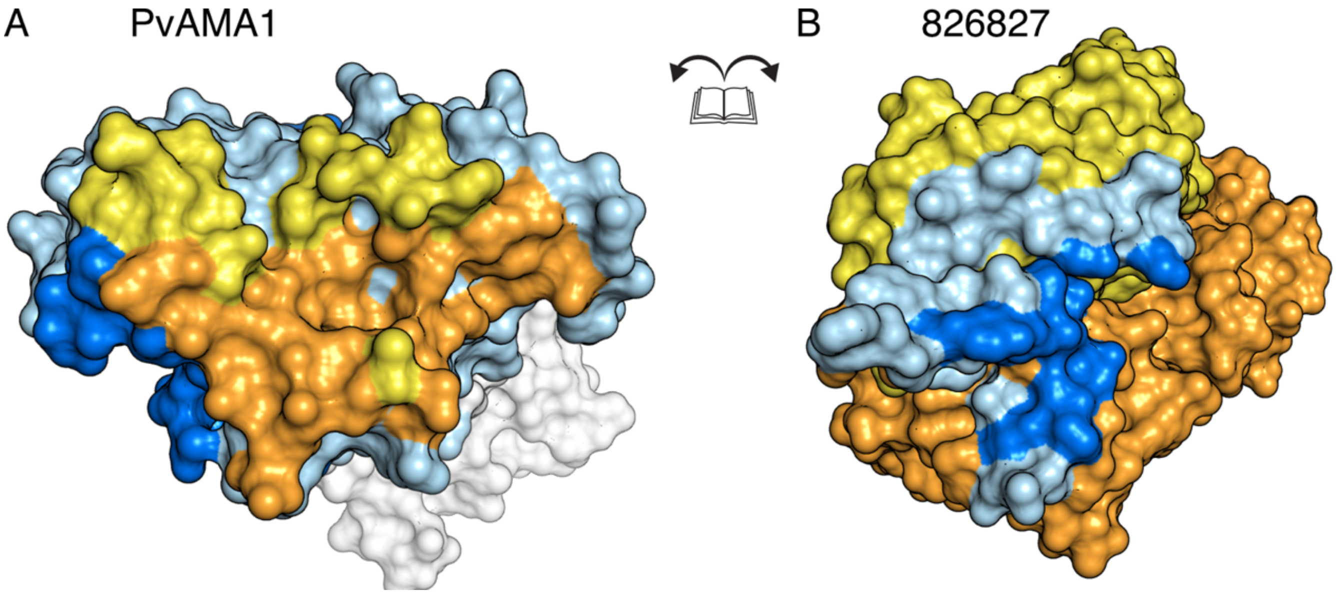

Supplementary Figure 11. Representative 2Fo-Fc electron density map of the PvAMA1-826827 crystal structure. The final 2Fo-Fc electron density map (blue, contoured at 1.0 σ) of CDR-H3 residues 105-121 (A), and PvAMA1 residues 308-317 of the D2-loop (B).

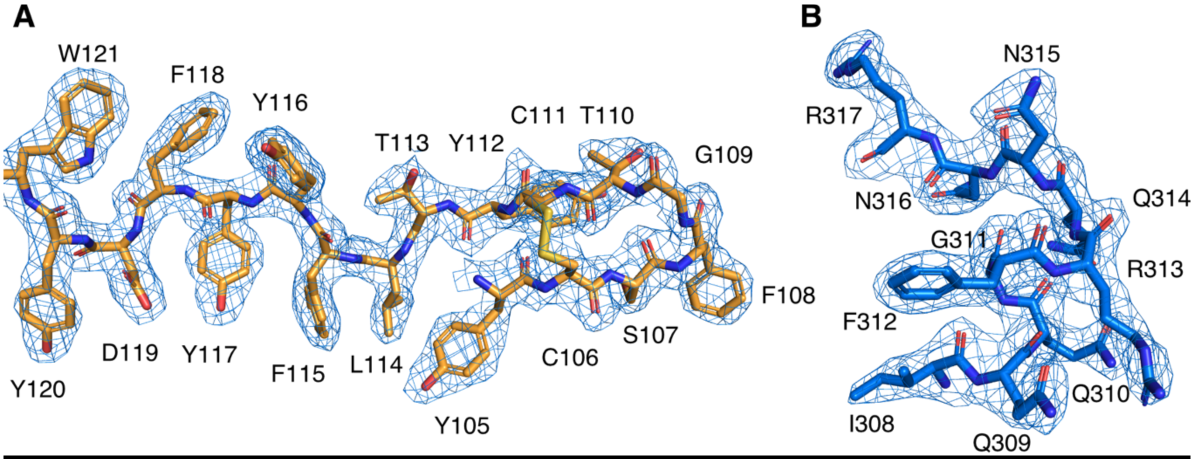

Supplemental Table 1 – VDJ alleles and CDR3 sequences and clonal groups

Shown are VDJ alleles and CDR3 sequences for 157 sorted and sequenced Bc IgH. The first column shows B cells that have been made into monoclonal antibodies, assigned a number, and the number of B cells in the clonal group.

# in Clonal Group	HumAb (if produced)	V-GENE & allele	D-GENE & allele	J-GENE & allele	CDR3
1		IGHV4-30-4	IGHD3-10	IGHJ4	CARGSYIRPFDYW
3		IGHV4-31	IGHD1-26	IGHJ4	CARRSGNYYDYFDYW
		IGHV4-31	IGHD1-26	IGHJ4	CARRSGNYYDYFDYW
		IGHV4-31	IGHD1-26	IGHJ4	CARRSGNYYDYFDYW
2		IGHV4-31	IGHD4-11	IGHJ5	CAREDRNYGRSCFDYW
	**806807**	**IGHV4-31**	**IGHD4-11**	**IGHJ5**	**CAREDRNYGKSCFDYW**
1		IGHV4-39	IGHD5-12	IGHJ5	CARQQVSAYHSRPGWFDPW
1		IGHV4-39	IGHD5-24	IGHJ4	CARQGRGGYDWRPRGPDYW
2		IGHV4-31	IGHD3-22	IGHJ5	CARASSYYYDSSGYYYNWFDPW
		IGHV4-31	IGHD3-22	IGHJ5	CARASSYYYDSSGYYYNWFDPW
4		IGHV4-61	IGHD2-15	IGHJ4	CARAHGEGHCSGGSCYSLFYYFDYW
		IGHV4-61	IGHD2-15	IGHJ4	CARARGEAYCSGGTCYTLFYYFDYW
		IGHV4-61	IGHD2-15	IGHJ4	CARAHGEGYCSGGSCYSLFYYFDYW
		IGHV4-61	IGHD2-15	IGHJ4	CARAYGEGYCSFGTCYTLFYYFDNW
7		IGHV4-61	IGHD2-15	IGHJ4	CARGHGEGYCSGGTCYSLFYYFEYW
		IGHV4-61	IGHD2-15	IGHJ4	CARGHGEGYCSGGTCYSLFYYFEYW
		IGHV4-61	IGHD2-15	IGHJ4	CARGRGEGYCSGGNCYTLFYYFDYW
		IGHV4-61	IGHD2-15	IGHJ4	CARSRGEGYCSFGTCYTLFYYFDYW
	**826827**	**IGHV4-61**	**IGHD2-15**	**IGHJ4**	**CARSRGEGYCSFGTCYTLFYYFDYW**
		IGHV4-61	IGHD2-15	IGHJ4	CASGNGEGYCSGGTCYSLFYYFDYW
		IGHV4-61	IGHD2-15	IGHJ4	CVSANGEGYCSGGTCYSLFYYFDYW
1	**830831**	**IGHV4-30-4**	**IGHD2-21**	**IGHJ6**	**CARSVGDGYCSGGACFFLYYTGLDVW**
4		IGHV3-15	IGHD2-21	IGHJ6	CTTNVRSATGYYGLAVW
		IGHV3-15	IGHD2-21	IGHJ6	CTTDVVSATGYYGMATW
		IGHV3-15	IGHD2-21	IGHJ6	CTTDVVSATGYYGMATW
		IGHV3-15	IGHD2-21	IGHJ6	CTTDVVSATGYYGMATW
1		IGHV3-15	IGHD1-26	IGHJ6	CTTDPVGATGYYGMGVW
6		IGHV3-15	IGHD1-26	IGHJ6	CTTDLVGTTGYYGMDVW
		IGHV3-15	IGHD1-26	IGHJ6	CTTDLEGATGYYGMDVW
		IGHV3-15	IGHD1-26	IGHJ6	CTTDLEGATGYYGMDVW
	**810811**	**IGHV3-15**	**IGHD1-26**	**IGHJ6**	**CATDLVGATGYYGLDVW**
	**800801**	**IGHV3-15**	**IGHD1-26**	**IGHJ6**	**CTTDLVGTTGYYGMDVW**
		IGHV3-15	IGHD1-26	IGHJ6	CATDLVGTTGYYGLDVW
1		IGHV3-15	IGHD5-18	IGHJ3	CATDVRTSTGYSGLDVW
1		IGHV3-15	IGHD3-16	IGHJ4	CVTDSPSPHDYIWGSYRYTTVYW
1		IGHV3-15	IGHD3-16	IGHJ4	CTTDRPSSNDYIWGSYHFDFDYW
2		IGHV3-15	IGHD3-16	IGHJ5	CTTDHPGHYDYVWQSYDTTFDYW
		IGHV3-15	IGHD3-16	IGHJ5	CTTDHPGHYDYIWGSYDLTVDSW
1		IGHV4-39	IGHD1-26	IGHJ4	CARHRRANNDFDYW
2	**816817**	**IGHV4-59**	**IGHD1-20**	**IGHJ5**	**CARETLNQRRFDLW**
		IGHV4-59	IGHD1-20	IGHJ5	CARETINQRRFEYW
1		IGHV5-10-1	IGHD3-9	IGHJ3	CARQVYDILNWYFDLW
2		IGHV4-59	IGHD5-12	IGHJ4	CARSLTRLRGFFGLFDYW
		IGHV4-59	IGHD5-12	IGHJ4	CARSLTRLRGFFGLFDYW
3		IGHV5-10-1	IGHD3-3	IGHJ4	CARRDFWSGYPYW
		IGHV5-10-1	IGHD3-3	IGHJ4	CARRDFWSGYPYW
		IGHV5-10-1	IGHD3-3	IGHJ4	CARRDFWSGYPYW
1		IGHV5-10-1	IGHD2-8	IGHJ3	CGRLSSTWGVLDIW
1		IGHV5-10-1	IGHD3-10	IGHJ4	CARWDGSGSYFGYW
1		IGHV5-10-1	IGHD4-17	IGHJ3	CARRTLTRDAFDIW
15		IGHV5-10-1	IGHD2-15	IGHJ4	CARLSSTWYAVDNW
		IGHV5-10-1	IGHD2-15	IGHJ3	CARLSLTWGVFDVW
		IGHV5-10-1	IGHD2-15	IGHJ3	CARLSLTWGVFDIW
		IGHV5-10-1	IGHD2-15	IGHJ3	CARLSLTWGVFDIW
		IGHV5-10-1	IGHD2-15	IGHJ3	CARLSLTWGVFDIW
		IGHV5-10-1	IGHD2-15	IGHJ3	CARLSLTWGVFDIW
		IGHV5-10-1	IGHD2-15	IGHJ3	CARLSLTWGAFDIW
		IGHV5-10-1	IGHD2-15	IGHJ3	CARLSLTWGAFDIW
		IGHV5-10-1	IGHD2-15	IGHJ3	CARLSLTWGAFDIW
		IGHV5-10-1	IGHD2-15	IGHJ3	CARLSLTWGAFDIW
		IGHV5-10-1	IGHD2-15	IGHJ3	CARLSLTWGAFDIW
		IGHV5-10-1	IGHD2-15	IGHJ3	CARLSLTWGAFDIW
		IGHV5-10-1	IGHD2-15	IGHJ3	CARLSLTWGAFDIW
		IGHV5-10-1	IGHD2-15	IGHJ3	CARLSLTSGVFDIW
		IGHV5-10-1	IGHD2-15	IGHJ3	CARLSLTSGVFDIW
1		IGHV5-10-1	IGHD1-20	IGHJ4	CARHSSLWNAIDSW
2		IGHV5-10-1	IGHD2-21	IGHJ6	CARASLTYYGMDVW
		IGHV5-10-1	IGHD2-21	IGHJ6	CARASLTYYGMDVW
5		IGHV3-11	IGHD4-11	IGHJ5	CARALEYANTWYPYW
		IGHV3-11	IGHD4-11	IGHJ5	CARALEYANTWYPYW
		IGHV3-11	IGHD4-11	IGHJ5	CARALEYANTWYPYW
		IGHV3-11	IGHD4-11	IGHJ5	CARALEYANTWYPYW
		IGHV3-11	IGHD4-11	IGHJ5	CARALDYANTLYPLW
2		IGHV3-74	IGHD5-23	IGHJ4	CARAGHVYSPNFDYW
		IGHV3-74	IGHD5-24	IGHJ4	CARAGHTYSPNFDYW
2		IGHV5-10-1	IGHD3-10	IGHJ4	CARVSLVRGVILDYW
		IGHV5-10-1	IGHD3-10	IGHJ4	CARMTMVRGVILDYW
1		IGHV5-10-1	IGHD3-3	IGHJ3	CARLTLLGGNAFDIW
1	**832833**	**IGHV1-3**	**IGHD2-21**	**IGHJ3**	**CAREYCDTGRCLAGVVVIPAEAFDIW**

# in Clonal Group	HumAb (if produced)	V-GENE & allele	D-GENE & allele	J-GENE & allele	CDR3
3		IGHV5-10-1	IGHD3-10	IGHJ6	CARLSMVQGGGIDVW
		IGHV5-10-1	IGHD3-10	IGHJ6	CARLSMVQGGGIDVW
		IGHV5-10-1	IGHD3-10	IGHJ6	CARLSMVQGGAIDVW
4		IGHV5-10-1	IGHD3-10	IGHJ3	CARITMTWGGAFEIW
		IGHV5-10-1	IGHD2-8	IGHJ3	CARITLTWGGTFDFW
	**838839**	**IGHV5-10-1**	**IGHD2-8**	**IGHJ3**	**CARITLTWGGTFDFW**
		IGHV5-10-1	IGHD1-26	IGHJ3	CARISLTWGGTLDIW
1	**804805**	**IGHV3-30-5**	**IGHD2-2**	**IGHJ4**	**CAKELYAYSTSPLDNW**
1		IGHV3-33	IGHD6-13	IGHJ5	CARGRGGQHESWFDPW
1		IGHV3-30	IGHD4-11	IGHJ6	CARDRLSATTVGYFEYW
4		IGHV3-30	IGHD3-9	IGHJ4	CAKSRPAYTLTAPFDSW
		IGHV3-30	IGHD3-9	IGHJ4	CAKSRPAYTLTAPFDSW
		IGHV3-30	IGHD3-9	IGHJ4	CAKNRPTYTLTAPFDSW
		IGHV3-30	IGHD3-9	IGHJ4	CAKNRPTYTLTAPFDSW
1		IGHV3-30	IGHD3-3	IGHJ4	CAKGRGSFTLTTPFDYW
1		IGHV3-30	IGHD4-17	IGHJ4	CAKDSGVATMGGAYHYW
1		IGHV5-10-1	IGHD5-18	IGHJ2	CARRGTSMPGDWYFDLW
6		IGHV5-10-1	IGHD3-3	IGHJ4	CARLSVVTFLSGFPDYW
		IGHV5-10-1	IGHD3-3	IGHJ4	CARLSVVTFLSGFPDSW
		IGHV5-10-1	IGHD3-3	IGHJ4	CARLSVVTFLSGFPDSW
		IGHV5-10-1	IGHD3-3	IGHJ4	CARLSVVTFLSGFPDSW
		IGHV5-10-1	IGHD3-3	IGHJ4	CARLSVVTFLSGFPDHW
		IGHV5-10-1	IGHD3-3	IGHJ4	CARLSVVTFLSGFPDHW
2		IGHV5-10-1	IGHD3-22	IGHJ4	CARLEGLYDSSGYFGYW
		IGHV5-10-1	IGHD3-22	IGHJ4	CARLEGLYDGSGHFGHW
5		IGHV5-10-1	IGHD3-10	IGHJ4	CARLAGSGTYELSFDYW
		IGHV5-10-1	IGHD3-10	IGHJ4	CARLAGSGTYELSFDYW
		IGHV5-10-1	IGHD3-10	IGHJ4	CARLAGSGTYDLSFDYW
		IGHV5-10-1	IGHD3-10	IGHJ4	CARLAGSGSYEFSFDSW
		IGHV5-10-1	IGHD3-10	IGHJ4	CARLAGSGSYEFSFDSW
1		IGHV5-10-1	IGHD3-10	IGHJ4	CARLAGSGTFAFSFDYW
3		IGHV5-10-1	IGHD3-10	IGHJ4	CARGLSYGSGTYYFDYW
		IGHV5-10-1	IGHD3-10	IGHJ4	CARGLSYGSGTYYFDSW
		IGHV5-10-1	IGHD3-10	IGHJ4	CARGLSYGSGSYYFDYW
1		IGHV1-46	IGHD6-19	IGHJ5	CARVYSSGWPPTKAFDPW
1		IGHV1-24	IGHD6-13	IGHJ4	CTTLRASGTFDSW
2	**808809**	**IGHV3-33**	**IGHD6-13**	**IGHJ4**	**CARDGLGYCLGGRCYPSLDYW**
		IGHV3-33	IGHD2-15	IGHJ4	CARNGLGYCLGGRWYPSLDYW
1		IGHV1-46	IGHD3-22	IGHJ4	CARGPAVFRACYDCSPSYTDYW
1		IGHV1-46	IGHD3-22	IGHJ4	CARGPAVERRFYDSIPSYPDYW
7		IGHV3-21	IGHD3-9	IGHJ5	CARGGGDILAGFYVSENWLDPW
		IGHV3-21	IGHD3-9	IGHJ5	CARGGGDILAGVYVSENWLDPW
		IGHV3-21	IGHD3-9	IGHJ5	CARGGGDILTGFYVSENWLDPW
		IGHV3-21	IGHD3-9	IGHJ5	CARGGGDILTGFYVSENWLDPW
		IGHV3-21	IGHD3-9	IGHJ5	CARGGGDILTGYYVSDNWLDPW
		IGHV3-21	IGHD3-9	IGHJ5	CARGGGDILTGYYVSENWFDPW
		IGHV3-21	IGHD3-9	IGHJ5	CVRGGGDILAGFYVSDNWLGPW
3		IGHV3-23	IGHD5-18	IGHJ2	CAKGWTREYTVYDHMLSHFDLW
		IGHV3-23	IGHD5-12	IGHJ2	CAKNWARVGTVYDPMLSHFDLW
		IGHV3-23	IGHD5-12	IGHJ2	CAKNWARVGTVYDPMLSHFDLW
5		IGHV3-23	IGHD3-3	IGHJ6	CAKDLAPPEFWSSYSYYGMDVW
		IGHV3-23	IGHD3-3	IGHJ6	CARDLAPPDFWSSYSYYGMDVW
		IGHV3-23	IGHD3-3	IGHJ6	CARDLAPPDFWSSYSYYGMDVW
		IGHV3-23	IGHD3-3	IGHJ6	CARDLAPPEFWSSYSYFGLGVW
	**814815**	**IGHV3-23**	**IGHD3-3**	**IGHJ6**	**CARDLAPPEFWSSYSYFGLGVW**
1		IGHV3-23	IGHD3-3	IGHJ6	CARDSAPPEFRSSYSYFGLGVW
1		IGHV3-23	IGHD3-3	IGHJ6	CARGLGPPEFWSCNSYFGLGVW
1		IGHV3-43	IGHD3-10	IGHJ4	CAKDREFGFGELFYYYYGMDVW
1		IGHV1-3	IGHD2-2	IGHJ6	CAGERLGYCSGTSCYYYSGMDVW
3		IGHV1-3	IGHD2-15	IGHJ6	CAREQLGYCSGGSCYYYYGMDVW
		IGHV1-3	IGHD2-15	IGHJ6	CAREQLGYCSGTSCYYYYGMDVW
		IGHV1-3	IGHD2-15	IGHJ6	CARERLGYCSGSSCYYYYGMDVW
1		IGHV1-69	IGHD2-15	IGHJ4	CAREKFATYCSGDNCYGFNFDYW
1		IGHV5-51	IGHD4-11	IGHJ6	CARHGDYATYPSSLYNYYGMDVW
5		IGHV3-48	IGHD3-22	IGHJ6	CARVTYYYDSSAYRTDYYYAMDVW
		IGHV3-48	IGHD3-22	IGHJ6	CARLSYYYDSSAYRTDFYYAMDVW
		IGHV3-48	IGHD3-22	IGHJ6	CARVTYYYDSSAYRTDFYYNMDVW
		IGHV3-48	IGHD3-22	IGHJ6	CARLSYYYDSSAYRTDFYYAMDVW
		IGHV3-48	IGHD3-22	IGHJ6	CARLAYYYDTGAYRTDFYYAMDVW
1		IGHV3-48	IGHD3-22	IGHJ6	CARVMHFYDASAYRTDFYFTMDVW
2		IGHV3-69	IGHD3-22	IGHJ6	CARLAYYYDTGAYRTDFYYAMDVW
		IGHV3-69	IGHD3-22	IGHJ6	CARLAYNYDTGAYRTDFYYAMDVW
4		IGHV5-10-1	IGHD2-15	IGHJ6	CARLYYSGANCYSGGTYYYHVDVW
		IGHV5-10-1	IGHD2-15	IGHJ6	CARLYCSGVNCYSGGTYYYHVDVW
		IGHV5-10-1	IGHD2-15	IGHJ6	CARLYCSGANCYSGGTYYYHVDVW
		IGHV5-10-1	IGHD2-15	IGHJ6	CARLYCSGANCYSGGNYYYHVDVW
1		IGHV1-46	IGHD2-15	IGHJ6	CARDRSAGGGSWTGSLYHYYGLGVW
1	**828829**	**IGHV4-39**	**IGHD3-22**	**IGHJ5**	**CARRSAEGYCSGGSCYSLWGVGFGPW**

Supplemental Table 2 – Avidity Index (AI_{50}) of PvAMA1 specific humAbs to different AMA1 constructs

AI_{50}	800801	804805	806807	808809	810811	814815	816817	826827	828829	830831	832833	838839
PvAMA1_ Palo Alto	1.01	1.93	3.30	0.80	0.88	1.26	1.40	2.25	1.12	1.12	2.77	1.64
PvAMA1_ PNG16	0.96	2.57	2.93	1.04	0.87	0.76	0.93	2.08	0.78	0.53	3.00	1.43
PkAMA1	0.95	0.46	3.10	1.23	0.03	0.00	1.37	3.87	0.89	2.16	0.78	2.21

Supplemental Table 3 - List of SNP and haplotypes observed in AMA1 sequences in isolates tested in response to 826827

Haplotypes	Normalized inhibition by 826827 (100ug/ml)	D107	R112	G117	K120	N130	N132	L140	A141	E145	E189	K190	H193	P210	E227	S228	N238	V239	A276	E277	G288	K352	Q380	V382	L384	E385	R438
H1	54.34	A	TK	-	R	K	D	-	E	A	N	-	Y	S	V	D	-	-	-	K	-	-	-	-	PR	Q	H
H2	41.41	A	TK	R	R	K	D	I	-	A	NK	E	-		V	D	-	-	-	K	E	-	R	-	-	-	-
H3	51.5	A	T	-	R	-	-	-	E	A	-	-	-	-	-	-	K	L	P	K	E	-	R	-	-	-	-
H4	54.43	A	T	-	R	-	-	I	-	A	NK	E	-	-	V	D	-	-	-	K	E	-	R	E	P	-	H
H5	44.1	A	T	-	-	-	-	I	-	A	K	E	-	S	V	D	-	-	-	-	-	-	-	-	R	-	-
H6	91.3	-	K	-	R	-	-	-	-	A	-	-	-	S	-	-	-	-	-	K	E	-	-	E	P	-	H
H7	88.97	A	T	-	R	K	D	-	-	-	N	-	-	S	-	-	-	-	-	-	-	-	-	-	R	-	H

Supplemental Table 4 – Crystallography data collection and refinement statistics

	PvAMA1-Fab 826827
PDB ID	9DX6
**Data collection statistics**	
Wavelength (Å)	0.953725
Space group	C121
Cell axes (Å) (a, b, c)	187.24, 54.46, 104.13
Cell angles (º) (α, γ, β)	90, 97.20, 90
Resolution range (Å)	47.76-2.40 (2.54-2.40)*
Completeness (%)	99.9 (99.8)
Total no. of reflections	289692 (45358)
Unique reflections	41378 (6614)
Redundancy	7.0 (6.9)
R_{meas} (%)	18.0 (117.6)
$CC_{1/2}$ (%)	99.5 (64.2)
I/σ	9.53 (1.55)
Wilson B ($Å^2$)	42.79
**Refinement statistics**	
R_{work}/R_{free} (%)	19.2/ 23.5
No. of atoms	
Protein	6395
Water	259
B factors ($Å^2$)	
Chain A	46.8
Chain B	45.3
Chain C	49.6
Water	45.7
R.m.s. deviations	
Bond lengths (Å)	0.004
Bond angles (º)	0.668
Validation	
Ramachandran plot	
outliers (%)	0.0
favored (%)	96.6
Rotamer outliers (%)	0.9
C-beta outliers	0
MolProbity score	1.49

* The values in parentheses represent the highest-resolution shell.

Supplemental Table 5 - Interactions PvAMA1 – Fab 826827 based on PISA

PvAMA1	Group	826827	Location	Group	Distance
Hydrogen bonds					
Glu 83	OE2	Tyr 54	CDR-H2	OH	2.4
Glu 83	OE2	Tyr 55	CDR-H2	OH	3.3
Thr 116	OG1	Tyr 49	CDR-L2	OH	2.9
Asp 118	OD1	Gly 57	CDR-L2	N	3.0
Asp 118	OD2	Arg 54	CDR-L2	NH2	2.8
Asp 118	N	Arg 54	CDR-L2	O	2.9
Asp 118	O	Arg 54	CDR-L2	NH2	3.7
Ala 131	O	Lys 31	CDR-L1	N	2.9
Ala 131	O	Thr 32	CDR-L1	N	3.5
Asn 132	ND2	Thr 113	CDR-H3	O/N	2.8/ 3.5
Asp 133	N	Ser 30	CDR-L1	OG	2.9
Val 170	O	Cys 106	CDR-H3	N	2.7
Val 170	N	Cys 106	CDR-H3	O	2.7
Ala 172	N	Gly 104	CDR-H3	O	2.9
Tyr 196	OH	Arg 101	CDR-H3	NH2	3.5
Tyr 196	OH	Glu 103	CDR-H3	OE1/OE2	2.8/2.6
Gln 314	O	Tyr 34	CDR-H1	OH	3.6
Asn 315	ND2	Tyr 117	CDR-H3	OH	2.8
Asn 315	O	Arg 101	CDR-H3	NE	2.7
Asn 315	O	Tyr34	CDR-H1	OH	3.8
Asn 315	OD1	Arg 101	CDR-H3	NH2	3.6
Asn 316	ND2	Tyr 34	CDR-H1	OH	3.7
Lys 321	NZ	Tyr 105	CDR-H3	OH	2.6
Salt bridges					
Lys 321	NZ	Glu 103	CDR-H3	OE2	3.6
Asp 118	OD2	Arg 54	CDR-L2	NH2	2.8
Other interfacing residues in PvAMA1					
Asn 84	Gly 117	Gln 119	Phe 128	Pro 129	Asn 130
His 134	Ile 135	Arg 146	Tyr 147	Asn 150	Met 153
Thr 164	His 165	Ser 168	Phe 169	Met 171	Gly 173
Gln 175	His 181	Met 194	Gln 310	Arg 313	Arg 317
Glu 318					
Other interfacing residues in 826 (heavy chain)			Other interfacing residues in 827 (light chain)		
Ser 331	Pro 32	Gly 33	Ser 28	Ser 50	Thr 53
Tyr 35	Arg 56	Arg 99	Ala 55	Ser 56	Val 58
Gly 102	Ser 107	Phe 108	Tyr 91	Asn 92	Trp 94
Cys 111	Tyr 112	Leu 114			
Phe 115	Asp 119	Tyr 120			
