# Supplementary material for: Potent AMA1-specific human monoclonal antibody against Plasmodium vivax Pre-erythrocytic and Blood Stages
Source: Nat Commun. 2024 Dec 4;15:10556. doi: 10.1038/s41467-024-53848-4 (PMC11618605; doi:10.1038/s41467-024-53848-4)
Supplement: Supplementary file 3 — Description of Additional Supplementary Files [file 41467_2024_53848_MOESM3_ESM.pdf]

Description of Additional Supplementary Files

File Name: Supplementary Movie 1
Description: **PvAMA1 bound Fab826827 compared to RON2** The electrostatic surface potential of PvAMA1 (8U9D) is displayed without 826827 bound to it, then the superimposed RON2 is shown in magenta followed by the 826 CD3 loop in green, showing a tight overlap in the binding site. Next the backbone of 826 and 827 are displayed to show our complete co-crystal structure.

File Name: Supplementary Movie 2
Description: **Polymorphisms within PvAMA1 in relation to Fab826827** The co-crystals structure of PvAMA1 with humAb 826827 is shown as an overview where PvAMA1 is represented as a solid surface and 826 in blue and 827 in yellow. The movie zooms towards three residues of interest namely D132, N130 and G117 which represent the wildtype amino acid residues and then these residues switch to the mutations observed in our clinical isolates as well as in the known sequences from other clinical isolates.

File Name: Supplementary Movie 3
Description: **Morph between open and closed conformation of AMA1 Domain 2 loop** A morph between PDB ID 6N87 and 8U9D is shown that focuses on the Domain 2 loop movement. The PfAMA1 structure 6N87 was superimposed onto 8U9D prior to generating the intermediate states for the moving Domain 2 loop.
