# Supplementary material for: Potent AMA1-specific human monoclonal antibody against Plasmodium vivax Pre-erythrocytic and Blood Stages
Source: Nat Commun. 2024 Dec 4;15:10556. doi: 10.1038/s41467-024-53848-4 (PMC11618605; doi:10.1038/s41467-024-53848-4)
Supplement: Supplementary file 7 — Reporting Summary [file 41467_2024_53848_MOESM7_ESM.pdf]

nature portfolio

Corresponding author(s): Jürgen Bosch, Christopher L. King

Last updated by author(s): Oct 15, 2024

Reporting Summary

Nature Portfolio wishes to improve the reproducibility of the work that we publish. This form provides structure for consistency and transparency in reporting. For further information on Nature Portfolio policies, see our Editorial Policies and the Editorial Policy Checklist.

Statistics

For all statistical analyses, confirm that the following items are present in the figure legend, table legend, main text, or Methods section.

n/a	Confirmed	
☐	☒	The exact sample size (*n*) for each experimental group/condition, given as a discrete number and unit of measurement
☐	☒	A statement on whether measurements were taken from distinct samples or whether the same sample was measured repeatedly
☐	☒	The statistical test(s) used AND whether they are one- or two-sided<br>*Only common tests should be described solely by name; describe more complex techniques in the Methods section.*
☒	☐	A description of all covariates tested
☒	☐	A description of any assumptions or corrections, such as tests of normality and adjustment for multiple comparisons
☐	☒	A full description of the statistical parameters including central tendency (e.g. means) or other basic estimates (e.g. regression coefficient) AND variation (e.g. standard deviation) or associated estimates of uncertainty (e.g. confidence intervals)
☐	☒	For null hypothesis testing, the test statistic (e.g. *F*, *t*, *r*) with confidence intervals, effect sizes, degrees of freedom and *P* value noted<br>*Give P values as exact values whenever suitable.*
☒	☐	For Bayesian analysis, information on the choice of priors and Markov chain Monte Carlo settings
☒	☐	For hierarchical and complex designs, identification of the appropriate level for tests and full reporting of outcomes
☒	☐	Estimates of effect sizes (e.g. Cohen's *d*, Pearson's *r*), indicating how they were calculated

Our web collection on statistics for biologists contains articles on many of the points above.

Software and code

Policy information about availability of computer code

Data collection	NA
Data analysis	NA

For manuscripts utilizing custom algorithms or software that are central to the research but not yet described in published literature, software must be made available to editors and reviewers. We strongly encourage code deposition in a community repository (e.g. GitHub). See the Nature Portfolio guidelines for submitting code & software for further information.

Data

Policy information about availability of data

All manuscripts must include a data availability statement. This statement should provide the following information, where applicable:

- Accession codes, unique identifiers, or web links for publicly available datasets
- A description of any restrictions on data availability
- For clinical datasets or third party data, please ensure that the statement adheres to our policy

Coordinates and structure factors of rPvAMA1_Palo Alto in complex with 826827 have been deposited in the Protein Data Bank (PDB) under PDB ID 9DX6. The data generated in this study for are provided in the Supplementary Information/Source Data file.

Research involving human participants, their data, or biological material

Policy information about studies with human participants or human data. See also policy information about sex, gender (identity/presentation), and sexual orientation and race, ethnicity and racism.

Reporting on sex and gender	NA
Reporting on race, ethnicity, or other socially relevant groupings	NA
Population characteristics	NA
Recruitment	NA
Ethics oversight	Sample collection in Cambodia was approved by the National Ethics Committee for Health Research of Cambodia (192NECHR). Written informed consent was obtained prior to blood collection.

Note that full information on the approval of the study protocol must also be provided in the manuscript.

Field-specific reporting

Please select the one below that is the best fit for your research. If you are not sure, read the appropriate sections before making your selection.

☒ Life sciences ☐ Behavioural & social sciences ☐ Ecological, evolutionary & environmental sciences

For a reference copy of the document with all sections, see nature.com/documents/nr-reporting-summary-flat.pdf

Life sciences study design

All studies must disclose on these points even when the disclosure is negative.

Sample size	at least N=3 but in most experiments higher N numbers were used when available. Each experiment was reproduced, except of the humanized mouse experiment as the 12 mice already cost 12x3600$.
Data exclusions	No data was excluded
Replication	Yes, and we do show replicates in our work
Randomization	randomization was not applicable
Blinding	analysis was performed blinded and revealed at the end

Reporting for specific materials, systems and methods

We require information from authors about some types of materials, experimental systems and methods used in many studies. Here, indicate whether each material, system or method listed is relevant to your study. If you are not sure if a list item applies to your research, read the appropriate section before selecting a response.

Materials & experimental systems

n/a	Involved in the study	
☐	☒	Antibodies
☐	☒	Eukaryotic cell lines
☒	☐	Palaeontology and archaeology
☐	☒	Animals and other organisms
☒	☐	Clinical data
☒	☐	Dual use research of concern
☒	☐	Plants

Methods

n/a	Involved in the study	
☒	☐	ChIP-seq
☐	☒	Flow cytometry
☒	☐	MRI-based neuroimaging

Antibodies

Antibodies used	All commercially used antibodies were verified by the vendor. Our own produced humAbs were sequence verified

Validation | Sequence verified our own mAbs

Eukaryotic cell lines

Policy information about cell lines and Sex and Gender in Research

Cell line source(s) | HEK293T for expression of mAbs

Authentication | ATCC provided fresh stock, not further confirmed

Mycoplasma contamination | negative by ATCC

Commonly misidentified lines (See ICLAC register) | none

Animals and other research organisms

Policy information about studies involving animals; ARRIVE guidelines recommended for reporting animal research, and Sex and Gender in Research

Laboratory animals | FRG-humHep mice C57Bl/6

Wild animals | NA

Reporting on sex | all female, not applicable for our studies

Field-collected samples | P. vivax clinical isolates from Thailand and Cambodia

Ethics oversight | P. vivax-infected blood samples were collected from patients under the approved protocol by the Ethics Committee of the Faculty of Tropical Medicine, Mahidol University (MUTM 2018-016). Sample collection in Cambodia was approved by the National Ethics Committee for Health Research of Cambodia (192NECHR). Written informed consent was obtained prior to blood collection.

Note that full information on the approval of the study protocol must also be provided in the manuscript.

Plants

Seed stocks | NA

Novel plant genotypes | NA

Authentication | NA

Flow Cytometry

Plots

Confirm that:

- [x] The axis labels state the marker and fluorochrome used (e.g. CD4-FITC).
- [x] The axis scales are clearly visible. Include numbers along axes only for bottom left plot of group (a 'group' is an analysis of identical markers).
- [x] All plots are contour plots with outliers or pseudocolor plots.
- [x] A numerical value for number of cells or percentage (with statistics) is provided.

Methodology

Sample preparation | Depending on purpose as described in our Methods section

Instrument | BD FACSCanto™ II , Attune NeXt, FACS Aria II

Software | FlowJo 10.9

Cell population abundance	lowest abundance in cell sorting, all other experiments high abundance
Gating strategy	Depending on the experiment as described in our Methods section

☒ Tick this box to confirm that a figure exemplifying the gating strategy is provided in the Supplementary Information.
